# A bibliometric research based on hotspots and frontier trends of denosumab

**DOI:** 10.3389/fphar.2022.929223

**Published:** 2022-09-19

**Authors:** Bolin Ren, Xiaolei Ren, Lu Wang, Chao Tu, Wenchao Zhang, Zhongyue Liu, Lin Qi, Lu Wan, Ke Pang, Cheng Tao, Zhihong Li

**Affiliations:** ^1^ Department of Orthopedics, The Second Xiangya Hospital, Central South University, Changsha, China; ^2^ Hunan Key Laboratory of Tumor Models and Individualized Medicine, The Second Xiangya Hospital, Central South University, Changsha, China

**Keywords:** denosumab, bibliometric and network analysis, RANKL, osteoporosis, biclustering analysis

## Abstract

Denosumab is a monoclonal antibody that targets and inhibits the osteoclast activating factor receptor activator for nuclear factor-κB ligand (RANKL). It has been widely used in the treatment of osteoporosis, giant cell tumors of bone, and in the prevention of malignant skeletal-related events (SREs). We collected the research results and related MeSH terms of denosumab from 2011 to 2021 through the Web of Science and PubMed, respectively. The literature was visualized and analyzed by CiteSpace and bibliometric online analysis platforms. The MeSH terms were biclustered using the Bibliographic Co-Occurrence Analysis System (BICOMB) and graph clustering toolkit (gCLUTO). The results show that the number of denosumab-related annual publications had increased from 51 to 215, with the United States leading and Amgen Inc. being the most influential in the past 10 years. Articles published in the Journal of Bone and Mineral Research had the highest total citations. Three scholars from Shinshu University in Matsumoto, Yukio Nakamura, Takako Suzuki, and Hiroyuki Kato, joined the field relatively late but produced the most. The clinical comparison and combination of denosumab with other drugs in the treatment of osteoporosis was the most significant focus of research. Drug withdrawal rebound and management strategies have gained more attention and controversy recently. MeSH analysis revealed eight major categories of research hotspots. Among them, exploring the multiple roles of the RANK-RANKL-OPG system in tumor progression, metastasis, and other diseases is the potential direction of future mechanism research. It is a valuable surgical topic to optimize the perioperative drug administration strategy for internal spinal fixation and orthopedic prosthesis implantation. Taken together, the advantages of denosumab were broad and cost-effective. However, there were still problems such as osteonecrosis of the jaw, severe hypocalcemia, a high recurrence rate of giant cells in the treatment of bone and individual sarcoidosis, and atypical femoral fractures, which need to be adequately solved.

## Introduction

Denosumab is a fully human IgG2 monoclonal antibody that targets and inhibits nuclear factor-κB ligand (RANKL) activators, an important driver of osteoclast differentiation and maturation. As an osteoprotegerin (OPG) variant, denosumab inhibits osteoclast differentiation by competitively binding to the RANKL factor with the receptor activator of nuclear factor-κB (RANK), a receptor on the osteoclast surface. With reduced RANK–RANKL binding, osteoclast formation, function, and survival are inhibited, bone resorption reduces, and bone mass increases (Thomas et al., 2010; Lacey et al., 2012). Notably, several orthopedics-related diseases are associated with RANK. Osteoporosis (OP) is characterized by an imbalance of bone remodeling and bone resorption over bone formation. It is a condition where bone mass decreases and bone microarchitecture is more susceptible to breaking due to increased fragility (Yasuda, 2021). Additionally, osteoclast-like multinucleated giant cells in giant cell tumor of bone (GCTB) express RANK, similar to osteoclasts in cell morphology, tissue differentiation, and regulation of bone resorption (Goldring et al., 1987). Bone fusion factors released by bone metastatic cancer cells can induce increased synthesis of RANKL by adjacent osteoblasts, resulting in an increased risk of skeletal-related events (SRE) due to dysregulation of osteoblasts/osteoclasts and bone destruction (Buijs and van der Pluijm, 2009). Furthermore, myeloma bone disease is characterized by regulation disorders among bone matrix, osteocytes, osteoblasts, osteoclasts, and immune cells. Osteocytes play an important role in bone loss by secreting factors, such as RANKL, sclerotin, and Wnt pathway inhibitor (DickkOP-1 protein) (Terpos et al., 2019). Therefore, denosumab has been used for the treatment of osteoporosis, giant cell tumor of bone that is unresectable or may cause severe impairment after surgery (Li H et al., 2020; Zhu et al., 2020), prevention of SREs in solid tumor bone metastases and multiple myeloma (MM), etc (Henry et al., 2011).

Bibliometrics is a comprehensive knowledge system in the era of big data with bibliography, statistics, and mathematics methods to quantitatively analyze knowledge carriers in a particular field in the form of statistics and inference. Authors, vocabularies, and the number of documents are the measurement objects to find the frontiers and focus (Ozsoy and Demir, 2018; Yang et al., 2021). CiteSpace is a software that uses the principle of bibliometrics to present the knowledge structure, rules, and distribution of a specific field in the form of a visual map (Synnestvedt et al., 2005; Chen et al., 2012). In addition, to make up for the shortcomings of traditional clustering and to control the overall and local information simultaneously, the Bibliographic Item Co-Occurrence Matrix Builder (BICOMB) was introduced to carry out a biclustering analysis. The graph clustering toolkit (gCLUTO) was used to extract the features of biclustering and present three-dimensional results (Wei et al., 2019). Although there has been a lot of research on denosumab in treating bone-related diseases, no bibliometric analysis has been performed to date. Therefore, we used various tools to systematically analyze denosumab’s application background and development trend in the past 11 years from different perspectives to evaluate focus and frontiers and provide decision information for future research.

## Methods

### Data collection

The data analyzed by CiteSpace and the online bibliometric platform (https://bibliometric.com/) were downloaded from the Web of Science (WOS). The retrieve strategy was TS = [((denosumab) OR (Prolia) OR (Xgeva)) AND ((bone tumors) OR (bone metastatic cancer) OR (osteoporosis) OR (skeletal-related Events) OR (bone disease))], and the retrieve time ranged from January 2011 to December 2021. Two thousand four hundred ninety documents were included regardless of the language and document types of the publications. All records and references were exported in plain text and tab-delimited file formats for the drawing and analysis of the scientific atlas. Subject headings and subheadings from the Medical Subject Headings (MeSH) have specificity, which can represent the main idea of the article. Furthermore, we set the same retrieve strategy and time range to collect the MeSH terms in PubMed literature and export them in XML format, which can be used for BICOMB and gCLUTO to carry out biclustering analysis.

### Statistical analysis

**FIGURE 1:**
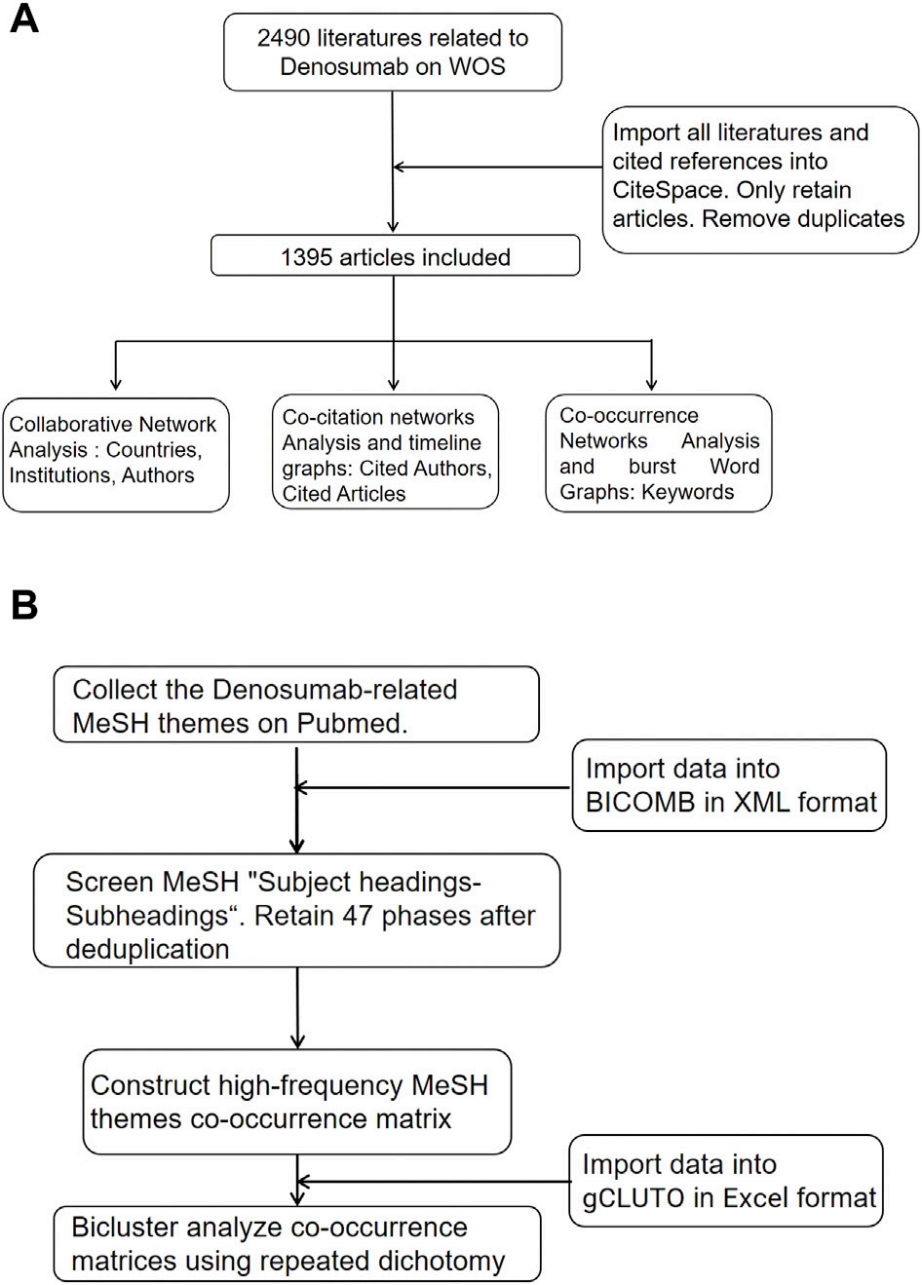
Flow chart of literature screening. **(A)** CiteSpace analysis flow chart. **(B)** MeSH themes biclustering analysis flow chart.

We imported the WOS data into CiteSpace (5.8 R2), retained only articles of the document type, and included 1,395 articles after removing duplicates (Figure 1A). The cooperative network analysis was performed to analyze countries, institutions, and authors. The co-citation analysis was performed to analyze cited journals and literature, and the conduct co-occurrence and burst detection were performed to analyze keywords (Ren et al., 2021). The area of the node represented the frequency of publication or citation. The colors from inside to outside and from cold to warm represented different years from 2011 to 2021, and the lines represented collaboration, co-citation, and co-occurrence relationships (Li R et al., 2020). BICOMB was developed by Cui et al. from the Department of Information Management and Information System (Medical) at China Medical University. Major MeSH terms could be exported to represent the publication core and rank the occurring frequency with Microsoft Excel and Go-PubMed. The screening publications were analyzed and checked with a designed model in XML format to extract the main information in the beginning, extract high-frequency MesH headings of all literature on PubMed, and generate a visual co-occurrence matrix (Yang, Pei, Wen, Zhou, and Tao, 2021). Biclustering of co-occurrence matrices in Excel format was performed using gCLUTO (1.0) with repeated dichotomies. In the chessboard diagram, the dark blocks represent the combination of two pairs of Mesh phrases on the row and column with higher frequency, and the lines outside the chessboard represent the clustering relationship of MeSH phrases. We then converted the cluster diagrams into hill maps. The height and volume of the hill were proportional to the similarity within the group and the number of objects contained within the group, respectively. The hotter red the peak color was, the smaller the standard deviation within the group was (Figure 1B).

## Results

### Trend of annual volumes of publications

**FIGURE 2:**
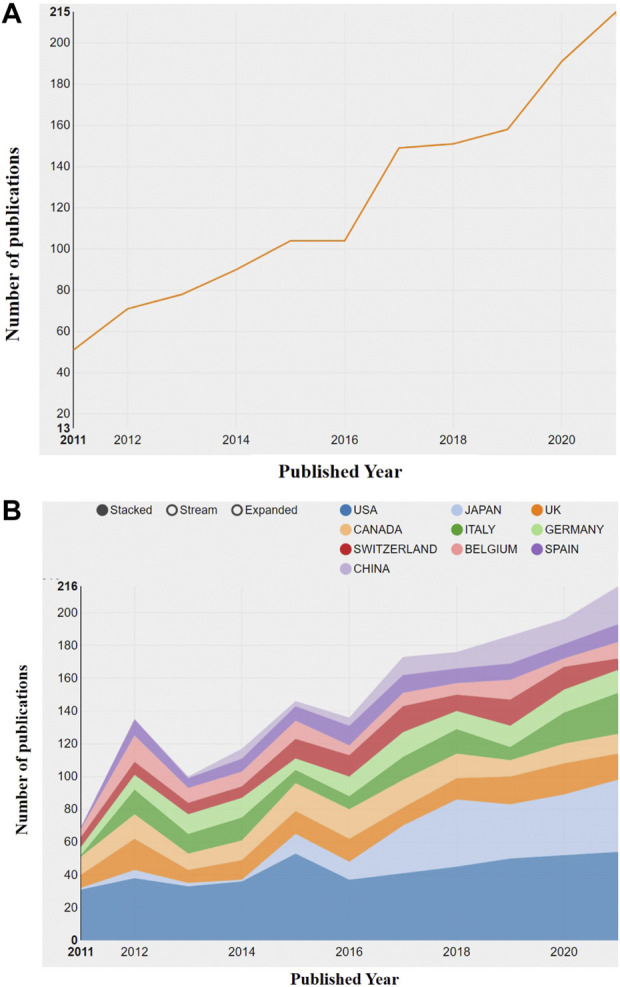
Visual diagram of annual publication changes. **(A)** Overview of annual publication changes of denosumab-related articles from 2011 to 2021. **(B)** Overview of annual publication changes in the top 10 countries in the field of denosumab from 2011 to 2021.

The WOS data were imported into CiteSpace (5.8 R2). After removing duplicates, only retained and 1,395 articles were included for statistical and quantitative analysis. On average, the citation number of each article was 23.39, and the overall H-index was 81. In the past 10 years, the annual publication number has continued to increase from 51 to 215 (Figure 2A). After two rounds of yearly output plateaus, two rapid growth stages followed. The former was related to the research hotspots of the impact of delayed use or of the use of denosumab on fracture risk of OP patients in 2017 (Anastasilakis et al., 2017a; McClung et al., 2017), while the latter was attributed to a new problem during the COVID-19 pandemic in 2019–2021, that is, the formulation of the transitional treatment plan for OP patients who received denosumab treatment in the COVID-19 era (Girgis and Clifton-Bligh, 2020; Yu et al., 2020). With the increasing use of denosumab in different regions, the attention will continue to increase in the future, and the number of publications is expected to maintain growth.

### Distribution characteristics of countries and institutions

**FIGURE 3:**
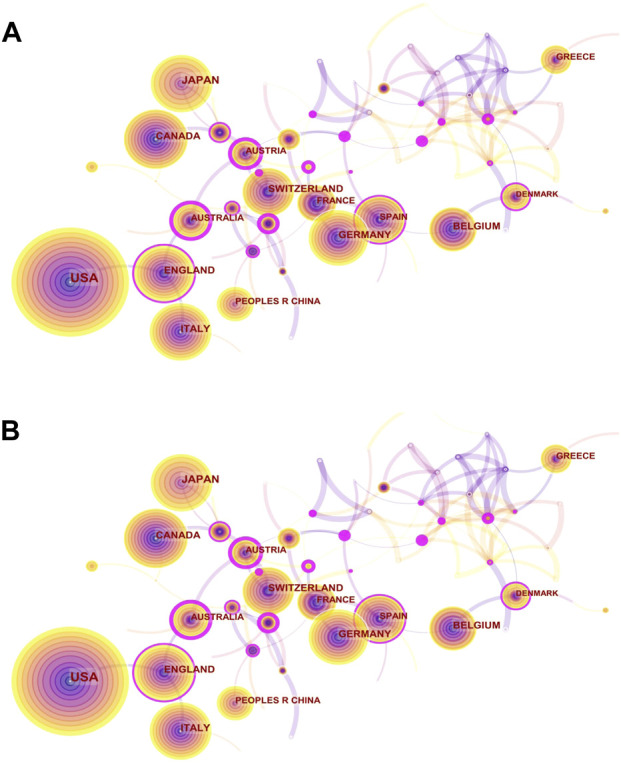
Visual network diagram of country and institution. **(A)** Country map of the denosumab study in 2011–2021. Nodes represent countries or regions, and lines represent cooperation. The colored rings in the nodes represent different years, and the purple outer circle represents higher centrality. **(B)** Network map of institutional cooperation in denosumab from 2011 to 2021. Nodes represent institutions, and lines represent cooperation. Colors in the nodes represent different years. The circle area represents the publication volume, and the purple outer circle represents higher centrality.

**FIGURE 4:**
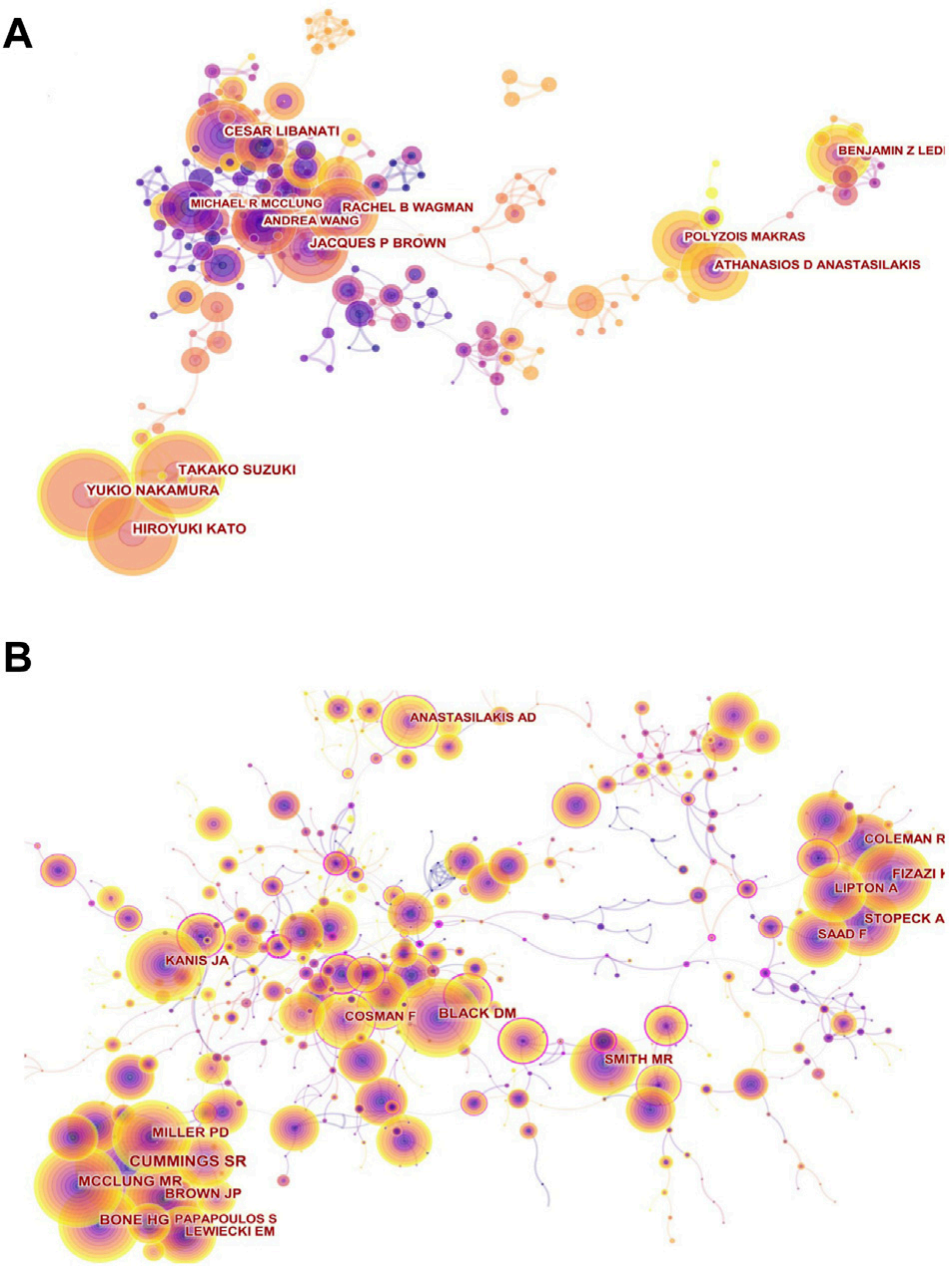
Visual network diagram of authors and cited authors. **(A)** Authors’ collaboration network map in denosumab from 2011 to 2021. The nodes represent authors. The lines represent cooperation. The different colors represent different years. **(B)** Co-citation network of cited authors in the denosumab field from 2011 to 2021. The nodes represent co-cited authors, and the lines represent co-citation relationships. Different colors in the nodes represent different years. The circle area represents the publication volume, and the purple outer circle represents higher centrality.

We presented the changes in the annual publication volume of the top ten countries (Figure 2B). The publications of international cooperation were regarded as the outputs of all cooperating countries in this chart, so the total number of annual publications in Figure 4 is larger than that in Figure 3. The national co-occurrence graph showed 70 nodes and 320 connections (Figure 3A), demonstrating 1,395 articles published in 70 countries. We found that Japan and China have had the highest growth rate in the past 10 years, despite the small number of publications in the early stage. The total number of publications between Figure 4 and Figure 3 was quite different in 2012 and 2015, which inferred that the countries with high-publication volumes cooperated relatively frequently in those years. When analyzing the total publication volume and the H-index, we found that the United States was the most influential in this field with 453 (32.9%)/71, followed by Canada with 144 (10.32%)/49 and England with 132 (9.46%)/49. The top three countries with average citations are France (96.01), Belgium (75.64), and Germany (65.06) from the European Union, which had a high quality of literature. The top three centrality rankings were Portugal (0.46), Wales (0.40), and the Czech Republic (0.32) (Table 1), while the centrality of high-publication countries was generally low. It can be seen that there is close cooperation in some parts of Europe and Asia but a lack of cross-continental collaboration between big countries.

**TABLE 1:** Top 10 countries and regions in terms of publication volume and centrality.

Rank	Country	Publications	% of 1395	Average citations	H-index	Rank	Country	Centrality
1	United States	453	32.9	49.97	71	1	Portugal	0.46
2	Japan	185	13.26	19.4	25	2	Wales	0.4
3	Canada	144	10.32	60.4	49	3	Czech Republic	0.32
4	England	132	9.46	60.88	49	4	Israel	0.32
5	Italy	130	9.32	44.48	49	5	Australia	0.29
6	Germany	120	8.60	65.06	28	6	Sweden	0.29
7	Switzerland	114	8.17	53.09	35	7	Saudi Arabia	0.27
8	Belgium	94	6.74	75.64	38	8	Austria	0.23
9	Spain	90	6.45	54.92	28	9	South Africa	0.22
10	People’s Republic of China	77	5.52	19.4	16	10	Estonia	0.21

The institutions’ distribution graph contained 402 nodes and 1,108 links (Figure 3B). The top five institutions in terms of volume of publications regarding denosumab were Amgen Inc (158), Columbia Univ (43), Univ Sheffield (38), Univ British Columbia (34), and Massachusetts Gen Hosp (29). Notably, three of the top five are from the United States, and the other two are from Canada and the United Kingdom, lacking direct cooperation with each other and were consistent with national distribution features (Table 2). As the development institution of denosumab, Amgen Inc. was the most important institution for denosumab research, having a wide range of research topics regarding denosumab and whose publication volume was much higher than other institutions. Columbia Univ and Univ British Columbia have always focused on the clinical study of denosumab in treating OP and have made profound and comprehensive achievements in this field. High intermediary centrality institutions like Merck & Co Inc (0.13), Stony Brook Canc Ctr (0.13), and Univ Libre Bruxelles (0.12) were key hubs for international collaboration between institutions promoting denosumab to prevent SREs (Table 2). Studies from these institutions have shown that denosumab was superior to zoledronic acid (ZA) in preventing SREs in patients with advanced solid tumor bone metastases. However, its stronger anti-resorptive effect was also associated with hypocalcemia (Body et al., 2015; Lipton et al., 2016). In addition, Altona Ctr Clin Res (0.12) and Australian Catholic Univ (0.13) acted as bridges among institutions that evaluated the efficacy and safety of medication for males with low bone mass density (BMD), as well as the risk and transitional measures of discontinuation after long-term treatment in the denosumab anti-osteoporosis treatment (Orwoll et al., 2012; McClung, Wagman, Miller, Wang, and Lewiecki, 2017).

**TABLE 2:** Top 10 institutions in terms of publishing volume and centrality.

Rank	Institution	Publications	Original country	Institution	Centrality	Original country
1	Amgen Inc	158	United States	Australian Catholic	0.13	Australia
2	Columbia Univ	43	United States	Merk & Co Inc	0.13	United States
3	Univ Sheffield	38	England	Stony Brook Canc Ctr	0.13	United States
4	Univ British Columbia	34	Canada	Univ Libre Bruxelles	0.12	BELGIUM
5	Massachusetts Gen Hosp	29	United States	Altoona Ctr Clin Res	0.12	United States
6	Oregon Osteoporosis Ctr	27	United States	Oregon Osteoporosis Ctr	0.11	United States
7	Harvard Med Sch	27	United States	New Mexico Clin Res & Osteoporosis Ctr	0.11	United States
8	Aarhus Univ Hosp	26	Denmark	Univ Lyon	0.11	France
9	Amgen Europe GmbH	26	Switzerland	Univ Auckland	0.1	New Zealand
10	Shinshu Univ	25	Japan	Ctr Clin & Basic Res	0.1	Denmark

### Distribution characteristics of authors and cited authors

The author collaboration network map comprised 452 authors and 1,167 collaboration links (Figure 4A). We presented the top ten authors by publication and citations (Table 3). It is worth noting that Yukio Nakamura, Takako Suzuki, and Hiroyuki Kato from the Department of Orthopedics of Shinshu University recently occupied the top position in both the publication volume and the intensity of burst through close cooperation. Through a randomized prospect study, Yukio Nakamura et al. have confirmed for the first time that denosumab combined with teriparatide or vitamin D supplementation with calcium could inhibit bone metabolism more efficiently than denosumab monotherapy and improve BMD of the lumbar spine and hip joints (Nakamura et al., 2017a; Nakamura et al., 2017b; Suzuki et al., 2018). In addition, they observed and affirmed the effect of denosumab on BMD in patients with osteogenesis imperfecta (OI) and rheumatoid arthritis complicated with osteoporosis (Nakamura et al., 2017c; Uehara et al., 2017). Cesar Libanati and Jacques P Brown, having the fourth most publication volume, represented scholars who have been engaged in anti-osteoporosis of denosumab earlier and laid a solid foundation for this field. By thoroughly evaluating the safety and efficacy of denosumab for postmenopausal women by means of multi-center, long-term (5 and 10 years), large-sample, phase III clinical trials, they suggest that denosumab could continuously improve BMD, reduce bone turnover markers (BTMs), and maintain low fracture rates without increasing the incidence of adverse events (Papapoulos et al., 2012; Bone et al., 2017). Athanasios D Anastasilaksi et al. achieved an increase in the publication volume by focusing on hotspots and improving innovation. After comparing serum biochemical indexes among postmenopausal women with vertebral fractures (Dmab/Fx+, *n* = 5) and without vertebral fractures (Dmab/Fx-, *n* = 5) after discontinuation of denosumab and those with vertebral fractures that had not been treated with denosumab (Fx+, *n* = 5), they found that miR-503 and miR-222-2, which inhibit osteoclastic genesis and activity, were significantly lower in the (Dmab/Fx+) group than in the (Fx+) group. The upregulation of the downstream target genes RANK (13-fold) and CTSK (2.6-fold) mRNA was negatively correlated with miR-503 and miR-222-2. The index changes of the (Dmab/Fx-) group were the same as those of the (Dmab/Fx+) group. Thus, the upregulation of osteoclastic genesis and activation in patients with drug withdrawal was a key factor in increasing vertebral fragility (Anastasilakis et al., 2017a; Anastasilakis et al., 2017b).

**TABLE 3:** Top 10 authors in terms of publication volume and the top 10 most-cited authors.

Rank	Author	Publications	Total number of citations	Corresponding author	Cited author	Citation counts
1	Yukio Nakamura	24	124	7	Cummings SR	521
2	Takako Suzuki	23	123	7	Bone HG	310
3	Hiroyuki Kato	22	124	2	Black DM	310
4	Cesar Libanati	19	1,156	17	Miller PD	258
5	Jacques P Brown	19	1,773	7	Fizazi K	241
6	Rachel B Wagman	18	1,066	16	Brown JP	208
7	Athansios D Anastasilakis	17	384	5	Kanis JA	204
8	Polyzois Makras	16	381	4	Stopeck AT	179
9	Benjamin Z Leder	16	481	9	Smith MR	177
10	Andrea Wang	16	1,122	13	Lipton A	174

The analysis of articles by high-frequency co-cited authors Cummings SR, Bone HG, and McClung MR revealed that romosozumab was highly concerned (Figure 4B). Romosozumab could continuously reduce BTM as effectively as denosumab and improve BMD at multiple sites even more significantly than the latter (LS = 15.1 vs. 6.4%; TH = 5.4 vs. 3.6%, for 24 months) (Bone et al., 2011; McClung et al., 2014). With romosozumab treatment, the bone formation marker P1NP level showed a transient increase phase and then gradually dropped below the baseline at month 12. Both drugs showed reversible effects and the potential to replace each other (Bone, Bolognese, Yuen, Kendler, Miller, Yang, Grazette, San Martin, and Gallagher 2011; McClung et al., 2018).

### Analysis of cited journals and cited references

**FIGURE 5:**
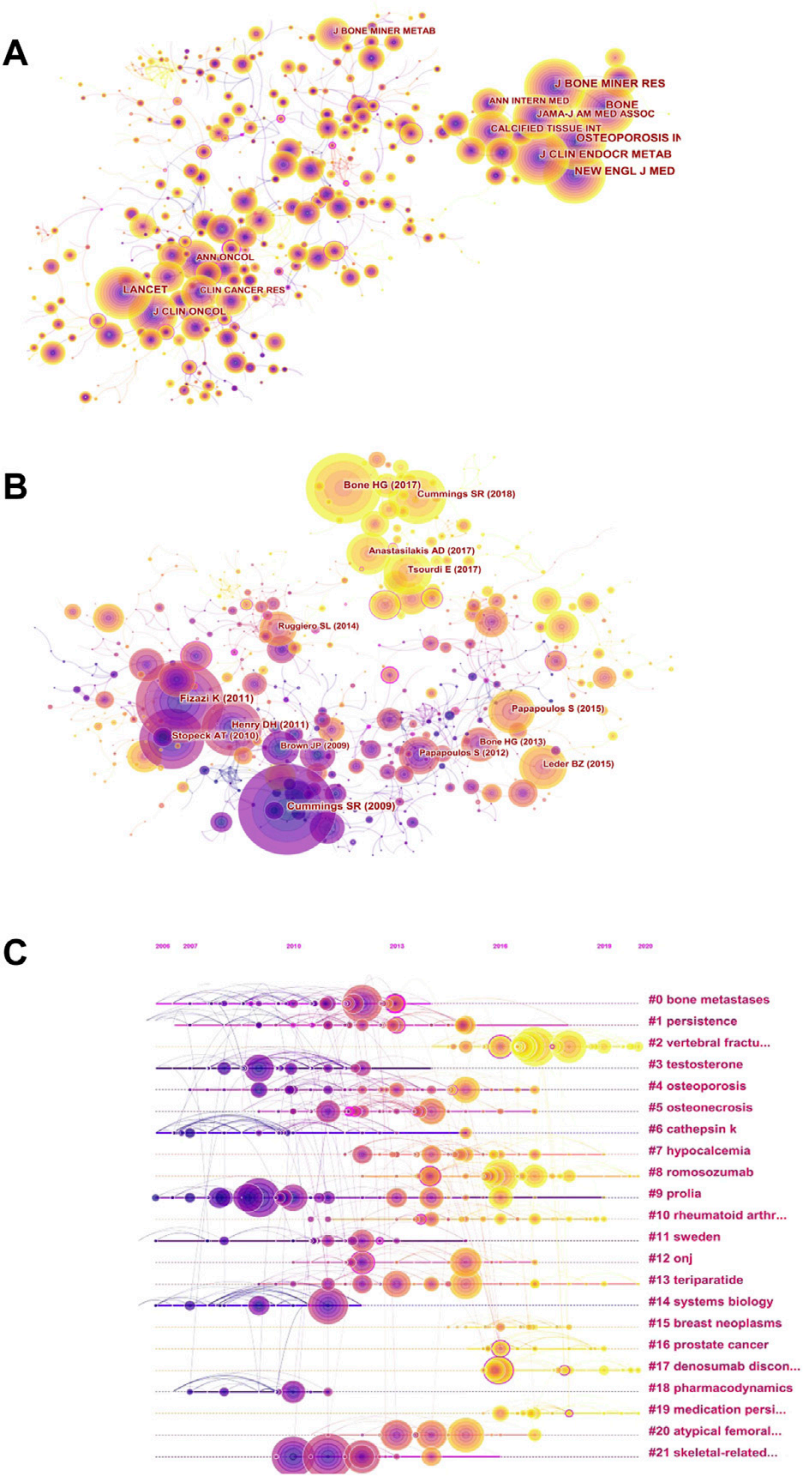
Visual network diagram of co-citation. **(A)** Co-citation network of cited journals in denosumab from 2011 to 2021. The nodes represent cited journals, and the lines represent co-citation relationships. Different colors in the nodes represent different years. The purple outer circle represents higher centrality. **(B)** Co-citation network of references in denosumab from 2011 to 2021. The nodes represent references, and the lines represent co-citation relationships. Different colors in the nodes represent different years. The purple outer circle represents higher centrality. **(C)** Timeline view based on the abstracts of the references in the denosumab field from 2011 to 2021. The nodes represent references, and the position on the horizontal line represents the year the reference was published. The lines represent co-citation relationships. Different colors in the nodes represent different years. The purple outer circle represents higher centrality.

To explore the background of the main modules of denosumab treatment, we performed a co-occurrence analysis of frequently cited journals, resulting in 571 nodes and 2,200 links (Figure 5A). The cluster analysis demonstrated that the frequently cited articles of the top five journals focused on fracture prevention and OP treatment caused by disorders of bone and mineral metabolism (Table 4). Several open-label multi-center or single-center clinical randomized controlled trials provided high-quality evidence for the evaluation of denosumab in treating postmenopausal osteoporosis (PMOP) and other osteopenia diseases. Among them, the *Journal of Bone and Mineral Research* had the highest total citations, and the research results had a long time span. The impact of drug transition regimens on BMD has been a hot topic in recent years, and an article in this journal pointed out that long-term (4–6 years) medication (not limited to postmenopausal women) followed by 1 year of ZA therapy did not prevent BMD loss caused by discontinuation of denosumab (Sølling et al., 2020). The *Journal of Clinical Endocrinology and Metabolism* reported that short-term (1-year) treatment followed by a year of alendronate was effective in maintaining BMD (only in postmenopausal women) (Kendler et al., 2020). *The Lancet* and *Journal of Clinical Oncology,* ranked 6th and 7th, reported many multi-center randomized, double-blind controlled studies, which demonstrated good academic performance in bone metastasis of prostate cancer and breast cancer, respectively. Furthermore, these provided more robust evidence for denosumab’s prevention of SREs. To sum up, multi-center large-sample research is of great significance, and it is worth strengthening cross-border cooperation between institutions.

**TABLE 4:** Top 10 cited journals in terms of citation frequency and centrality.

Rank	Cited journal	Total citations	Average citations	IF (JCR 2021)	Centrality	Cited journal
1	J BONE MINER RES	944	97	6.741/(Q1)	0.43	EXP MOL MED
2	NEW ENGL J MED	904	12	91.2/(Q1)	0.32	Circulation
3	OSTEOPOROSIS INT	816	145	4.507/(Q2)	0.32	CELL MOL LIFE SCI
4	BONE	756	81	4.398/(Q1)	0.29	EUR UROL
5	J CLIN ENDOCRINOL METAB	680	78	5.958/(Q1)	0.23	AM J CLIN NUTR
6	Lancet	594	14	79.321/(Q1)	0.22	ARTHRITIS RHEUM-ARTH
7	J CLIN ONCOL	402	26	44.544/(Q1)	0.19	ARTHRITIS RES THER
8	JAMA-J AM MED ASSOC	338	7	56.272/(Q1)	0.18	KIDNEY INT
9	CALCIFIED TISSUE INT	329	33	4.333/(Q2)	0.17	INT J CLIN ONCOL
10	ANN ONCOL	253	13	32.976/(Q1)	0.16	Menopause

The subject knowledge base can be obtained by analyzing the literature with high citation frequency and centrality presented by the cited reference network (Figure 5B). The top five references in citation had significant influence in popular fields, and their themes were consistent with highly cited journals (Table 5). High centrality references signified an important turning point in research. As OP researchers were increasingly concerned about specific risks, the most central RCT confirmed that denosumab did not affect fracture healing in postmenopausal women, even if treatment was performed in the initial stage of fractures (Adami et al., 2012). Earlier studies suggest that the fracture risk after denosumab discontinuation was similar to that of a placebo (Brown et al., 2013), while Aubry Rozier B et al. reported 3 patients without a history of fragility fractures who developed severe spontaneous vertebral fractures about 1 year after denosumab treatment discontinuation. Hence, researchers have paid attention to the rebound effect (Aubry-Rozier et al., 2016). Furthermore, scholars majoring in preventing or delaying SREs have begun to focus on the cost-effectiveness of denosumab and found that denosumab reduced SREs and increased quality-adjusted life-years (QALYs) compared with ZA (Stopeck et al., 2012). To track the focus changes over time, references were generated as a timeline view (Figure 5C). The focus of the recent hot trend is “vertebral fracture"#2, “romosozumab"#8, “denosumab discontinuation"#17, followed by “prostate cancer"#16, “medication persistence"#19, and “rheumatoid arthritis"# 10.

**TABLE 5:** Top 5 references in terms of citation frequency and centrality.

Rank	Frequency	References	Author and publication year	Centrality	References	Author and publication year
1	127	Denosumab for prevention of fractures in postmenopausal women with osteoporosis	Cummings SR (2009)	0.23	Denosumab treatment in postmenopausal women with osteoporosis does not interfere with fracture healing: results from the FREEDOM trial	Adami S(2012)
2	114	Denosumab versus zoledronic acid for treatment of bone metastases in men with castration-resistant prostate cancer: a randomized, double-blind study	Fizazi K (2011)	0.22	Cost-effectiveness of denosumab vs zoledronic acid for prevention of skeletal-related events in patients with solid tumors and bone metastases in the United States	Stopeck Alison(2012)
3	98	10 years of denosumab treatment in postmenopausal women with osteoporosis: results from the phase 3 randomized FREEDOM trial and open-label extension	Bone HG(2017)	0.21	Discontinuation of denosumab and associated fracture incidence: analysis from the fracture reduction evaluation of denosumab in osteoporosis every 6 months (FREEDOM) trial	Brown JP(2013)
4	85	Denosumab compared with zoledronic acid for the treatment of bone metastases in patients with advanced breast cancer: a randomized, double-blind study	Stopeck AT (2010)	0.17	Safety of long-term denosumab therapy: results from the open-label extension phase of two phase 3 studies in patients with metastatic breast and prostate cancer	Stopeck AT(2016)
5	79	Randomized, double-blind study of denosumab versus zoledronic acid in the treatment of bone metastases in patients with advanced cancer (excluding breast and prostate cancer) or multiple myeloma	Henry DH(2011)	0.16	Severe spontaneous vertebral fractures after denosumab discontinuation: three case reports	Aubry-Rozier B(2016)

### Analysis of keywords

**FIGURE 6:**
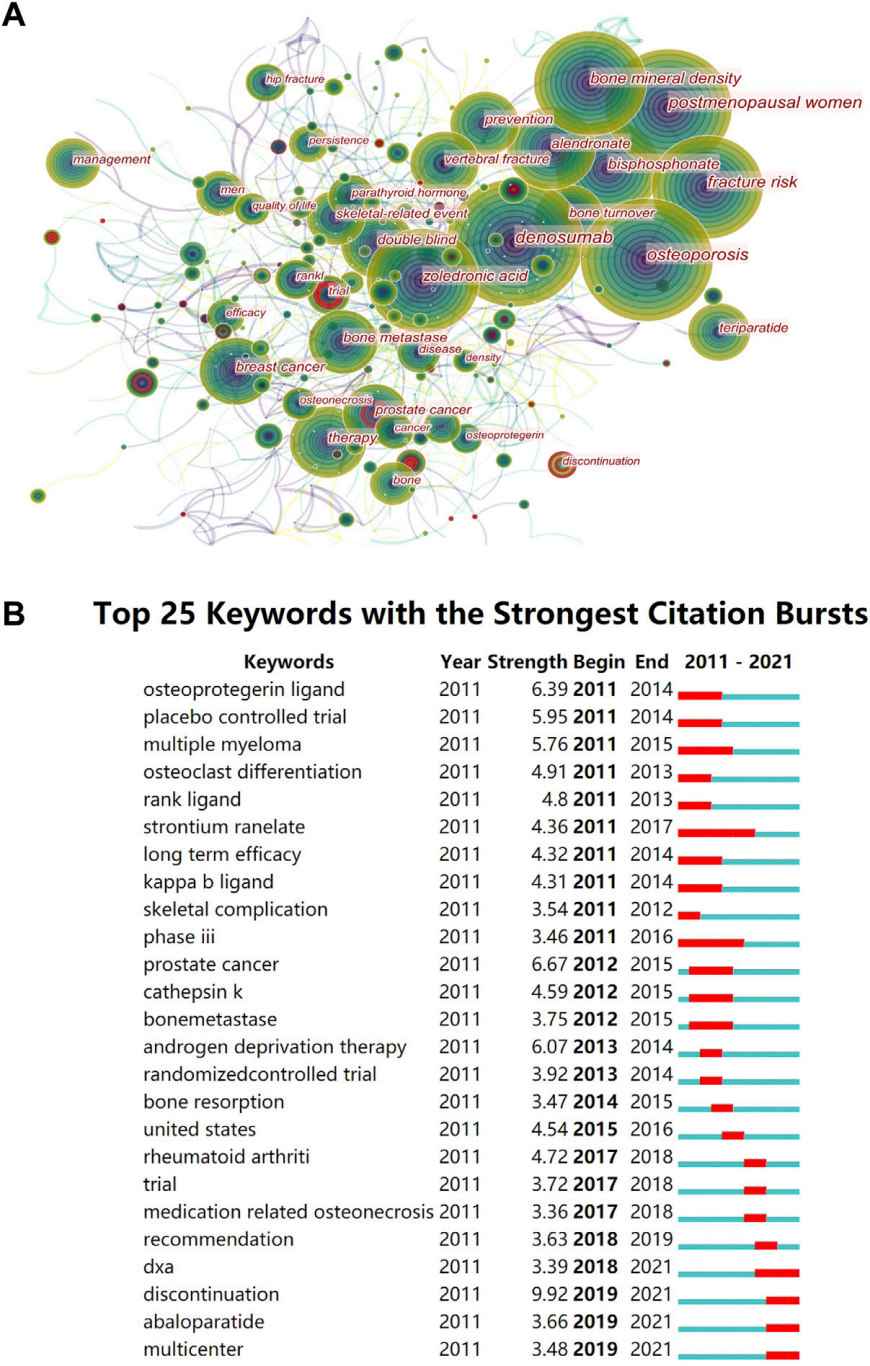
Visual diagram of keywords. **(A)** Keywords co-occurrence graph in denosumab from 2011 to 2021. The nodes represent keywords, and the lines represent co-occurrence. The red circle indicates that the keyword has a citation burst. The area represents the frequency of keyword occurrence. The purple outer circle represents higher centrality. **(B)** Top 25 keywords with the strongest citation bursts. The red bars are the years of burst duration.

The word frequency of keywords reflects the popularity of the topic. High centrality represents stronger characteristics and broader influence. Strong bursts are signs of emerging trends at a particular stage. The most popular topic in the keyword co-occurrence network was OP treatment (Figure 6A), and most high-frequency words were related to it (Table 6). The postmenopausal women were most concerned with the OP themes, and much of the research focused on comparing denosumab with ZA, alendronate, and teriparatide by monitoring BMD and BTM. Breast cancer and prostate cancer patients were the most prone to bone metastases and the most benefited groups of denosumab bone-targeted therapy. Adverse events in the long-term prevention of SREs in these patients were the second hot topic. Keywords with high centrality are “open-label,” “safety,” “exposure,” and “follow-up” (Table 6). High centrality analysis reflected that it was challenging to establish pure control and experimental groups in the clinical trials of denosumab due to complex factors, such as previous treatment experience, the severity of illness, and personal characteristics. Therefore, the open labels and long-term drug exposure plus follow-up were required to improve the confidence of the results and reveal potential safety issues.

**TABLE 6:** Top 15 keywords in terms of frequency and centrality.

Rank	Keyword	Frequency	Keyword	Centrality
1	Denosumab	828	Open-label	0.17
2	Osteoporosis	604	Safety	0.16
3	Postmenopausal women	567	Exposure	0.14
4	Fracture risk	406	Androgen deprivation therapy	0.13
5	Bone mineral density	347	Follow-up	0.12
6	Zoledronic acid	313	Biochemical marker	0.11
7	Bisphosphonate	298	Clinical trial	0.11
8	Alendronate	213	Bone histomorphometry	0.11
9	Prevention	172	Osteoporotic fracture	0.09
10	Breast cancer	161	Combination therapy	0.09
11	Bone metastases	160	Solid tumor	0.08
12	Double blind	156	Atypical femoral fracture	0.08
13	Vertebral fracture	153	Hip	0.08
14	Teriparatide	148	Cathepsin k inhibitor	0.08
15	Prostate cancer	130	Chemotherapy	0.08

We found that recent burst words included “discontinue,” “abaloparatide,” and “DXA” (Figure 6B). The “discontinue” term had the highest burst strength, and many trials were exploring the best solution to alleviate the rebound from denosumab discontinuation. Alendronic acid and romosozumab could avoid bone loss caused by denosumab discontinuation, but ZA was controversial, and teriparatide transiently exacerbated the disease (Yu, Tsourdi, Clarke, Bauer, and Drake, 2020). However, the rebound prevention effects of different denosumab courses, drug replacement timing, and dose need to be clarified further. Abaloparatide is a parathyroid hormone-related peptide, which is superior to teriparatide in improving cortical BMD and reducing the incidence of hypercalcemia. Currently, data on the combination of abaloparatide and denosumab or the prevention of denosumab withdrawal rebound are lacking, which is a potential new topic. Using regular DXA scans of the proximal femur in women receiving denosumab, nonvertebral fracture rates decreased with increasing total hip T-score but not with vertebral fracture or age (Ferrari et al., 2019). Therefore, obtaining dynamic T-scores by follow-up DXA could determine the future treatment aims of denosumab for OP.

### Research hotspots of denosumab based on MeSH term clusters

**FIGURE 7:**
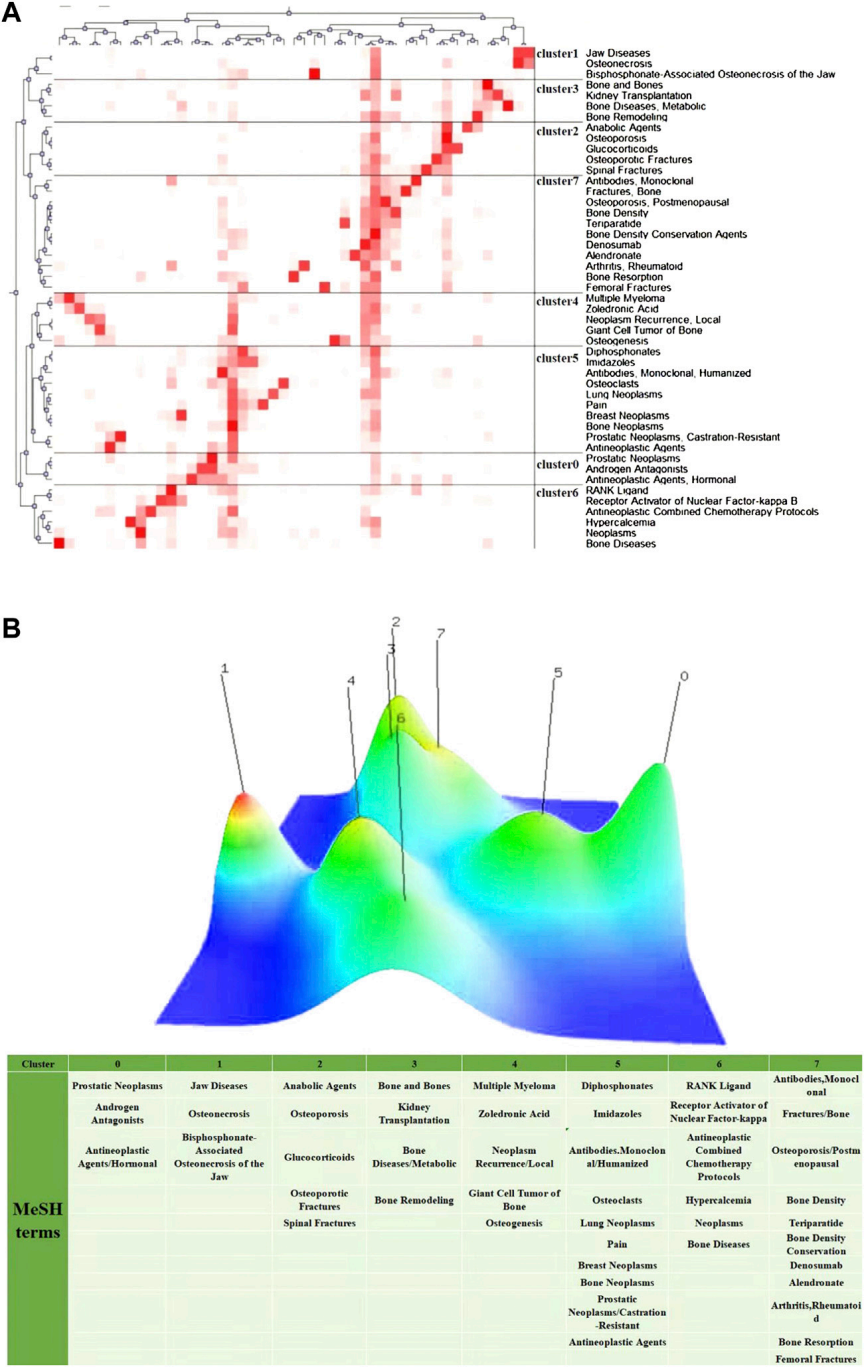
Visualization of biclustering analysis. **(A)** Matrix visualization of biclustering high-frequency major MeSH terms in denosumab from 2011 to 2021. **(B)** Mountain visualization of biclustering high-frequency major MeSH terms in denosumab from 2011 to 2021.

We collected all literature studies of the type “article” on PubMed from January 2010 to December 2021, which contained 209 “subject headings-subheadings” with a total frequency of 1,386 times. The MeSH terms that appeared four times or more were regarded as relatively high frequency. A total of 47 terms were screened out, with a total frequency of 1,167 times, accounting for 84.20% (1,167/1,386) (Supplementary Table S1). Among them, “Bone Density Conservation Agents” appeared 179 times, much higher than other terms, indicating that the major research objects were still denosumab and various BMD protection agents. The matrix visualization (Figure 7A) generated by biclustering analysis was divided into eight clusters, representing different hotspot directions. The results were converted into a hill map and displayed as 0 to 7 (Figure 7B). Four groups were summarized from the degree of similarity: (2, 7, 3), (4, 6), (0, 5), and (1), and each cluster is discussed in detail below.

## Discussion

### Application of denosumab in prostate tumors

Denosumab significantly delayed the onset of first bone metastases in prostate cancer [HR = 0.84 (0.71–0.98); *p* = 0.032], prolonged bone metastasis-free survival by an average of 4.2 months, and reduced bone-related events. In castration-sensitive prostate cancer patients, androgen receptor antagonists and hormone deprivation therapy led to high bone transition and rapid bone loss. Notably, denosumab can effectively improve BMD and prevent multiple fractures such as the spine. However, differences in treatment regimens significantly impact drug safety, with 120 mg subcutaneously every 4 weeks in the former being associated with higher rates of osteonecrosis of the jaw (5 vs. 0%) and hypocalcemia (2 vs. 0.28%). The incidence of adverse events in the latter with 60 mg every 6 months was not significantly different from placebo (Smith et al., 2009; Smith et al., 2012). To improve the safety of long-term treatment, it is worth establishing guidelines so that doctors can selectively provide therapeutic regimens according to the assessment and prediction of the patient’s condition. For example, a controlled study showed that males with a shorter prostate-specific antigen doubling time (PSADT) have higher risks of developing bone metastases or death and that denosumab treatment improved bone metastasis-free survival (BMSF) in males with a shorter PSADT (Smith et al., 2013) and had a more significant treatment effect in males at higher risk of progression. Therefore, treatment programs to prevent SRE should be started immediately for such patients.

### Denosumab-related complications of osteonecrosis of the jaw

Osteonecrosis of the jaw (ONJ) is closely related to long-term exposure to bone resorption inhibitors such as bisphosphonates (BP) and denosumab. Due to the high drug dose, its incidence in tumor patients was much higher than in OPs (1–15% vs. 0.001–0.01%) (Khan et al., 2015). Infection is a key cause of ONJ, and invasive dental treatment is a high-risk factor for infection. Ideally, dental treatment should be completed 2 weeks before initiating anti-bone resorptive treatment. However, studies showed that stopping BPs or denosumab before dental treatment in OP patients had no significant effect on the incidence of ONJ. Considering that the incidence of ONJ in OP treatment was low and the period of drug discontinuance may lead to decreased bone mineral density and increased fracture rate, the benefits of continuous use may outweigh the risks. Unlike the high-frequency administration for preventing SRE, denosumab only needs to be injected once every 6 months to treat OP. Therefore, patients have sufficient time to plan dental treatment during the 5-month interval after the drug’s half-life (Yoneda et al., 2017). It is worth noting that controlling the oral infection in tumor patients with previous ONJ properly avoids ONJ recurrence (Otto et al., 2015). Therefore, it is necessary to carry out oral hygiene education, avoid invasive dental treatment and oral infectious factors, and formulate individualized dosing regimens for cancer patients.

### Anti-OP treatment of denosumab in patients with anabolic agents

Glucocorticoid-induced osteoporosis (GIOP) is a serious adverse reaction caused by the long-term use of anabolic glucocorticoids, which is usually prevented and managed by bisphosphonates. Teriparatide, another anabolic agent, combined with denosumab, has been shown to increase lumbar spine and hip BMD more effectively than bisphosphonates (Saag et al., 2009; Saag et al., 2019). Notably, teriparatide shows an advantage in improving BMD in GIOP patients with bisphosphonate therapy history, especially in the femoral neck, compared with denosumab (Hirooka et al., 2020). This is likely because denosumab and bisphosphonates are anti-resorptive agents, while teriparatide is a pro-osteogenic agent (Hirooka et al., 2021). Perioperative drug therapy can improve bone microarchitecture in patients with osteoporotic spinal fractures and reduce the probability of internal fixation failure and loosening after pyramidal fusion (Bryant et al., 2021). Six months after the operation, the bone union rate (82 vs. 36%, *p* < 0.05) and spinal fusion rate (*p* < 0.05) scanned by CT were significantly higher in patients treated with teriparatide combined with denosumab than in teriparatide monotherapy (Ide et al., 2018). According to the abovementioned evidence, expanding the sample size and conducting further research to screen the combination drug strategy that can maximize the benefit of patients with GIOP and internal fixation surgery are necessary.

### Application of denosumab in kidney transplantation

Kidney transplant recipients often suffer from chronic kidney disease–mineral bone diseases (CKD-MBDs), which may develop into post-transplant bone diseases with different spectrums due to post-transplant immunosuppressants, renal function, and diabetes (Damasiewicz and Ebeling, 2017). Denosumab has superior efficacy to bisphosphonates and is not metabolized by the kidneys. The decrease in bone mineral density mainly occurs in the first year after transplantation, and the most apparent decrease (4–10%) occurs in the first 6 months (Bouquegneau et al., 2016). Hence, the use of bone-protective drugs should begin with CKD-MBDs before transplantation. However, studies have found a high incidence of severe hypocalcemia in patients with end-stage renal disease receiving denosumab (6/8 in CKD-5 and 2/5 in CKD-4) (Dave et al., 2015). Additionally, a retrospective analysis showed that kidney transplant recipients treated with denosumab were nearly twice as likely to develop urinary tract infections (cystitis) than the control group. The mechanism may be that the RANK/RANKL interaction is essential in developing dendritic cells and activated T lymphocytes expressing abundant RANKL. At the same time, denosumab interferes with the interaction, thus abolishing specific and non-specific immunity (Bonani et al., 2017; Zhang et al., 2020). Further data are needed to confirm the safety of denosumab in kidney transplant recipients, clarify its application with different renal functions, and develop treatment strategies for possible complications.

### Application of denosumab in multiple myeloma and giant cell tumor of bone

Multiple cells in bone tumors secrete RANKL, including myeloma and stromal cells in multiple myeloma (MM) and tumor stromal cells in giant cell tumor of bone (GCTB), while osteoclast-like multinucleated giant cells in GCTB express RANK, allowing denosumab to alter the tumor microenvironment during treatment. Denosumab was non-inferior to ZA in preventing MM-induced SRE and showed better efficacy in the Asian subgroup. Additionally, adverse events were similar except for hypocalcemia, which was higher in the denosumab group, making denosumab a better choice for patients who cannot tolerate ZA’s nephrotoxicity. However, the renal safety of denosumab has not been fully demonstrated, and clinical trials of the drug in patients with severe renal insufficiency (creatinine clearance <30 ml/min) are underway (Raje et al., 2018; Huang et al., 2020). Denosumab is the only drug approved for GCTB that is inoperable or requires neoadjuvant therapy. However, after treatment, studies have reported a high recurrence rate and individual sarcoma transformation. Studies have also found that denosumab fails to inhibit the proliferation of tumor stromal cells and the survival of osteoclasts. Aggressive surgery is needed to remove the tumor cells in the thickened new bone and reduce local recurrence. In addition, the dosage of denosumab should be adjusted for lifetime maintenance. Up to now, there have been three molecular hypotheses for GCTB sarcoma transformation involving RANKL, namely, the three hypotheses are immunosuppression, downregulation of nuclear factor IB (NKIB) level, and upregulation of the Sema3A gene, all of which are worthy of further exploration in basic scientific research (Müller et al., 2016; Chawla et al., 2019; Shibuya et al., 2019; Li H et al., 2020).

### The application of denosumab in other advanced cancers

The combined analysis found that denosumab delayed the onset of the first SRE by 8.2 months compared with ZA in the cancer types that are most prone to bone metastases and had a significant advantage in reducing the risk of multiple SREs (Lipton et al., 2012). In addition, denosumab was also approved for use in cancer patients with bone loss due to endocrine therapy and chemotherapy. Such patients need to assess the risk of bone loss early when using denosumab for anti-osteoporosis and be vigilant about drug discontinuation rebound (Dell'Aquila et al., 2020; Von Moos et al., 2019). Denosumab was superior to ZA in relieving bone pain caused by the action of osteoclasts. It was suggested that inhibiting the activity of osteoclasts was crucial for the observed pain relief. However, in bone metastases of lung cancer, the analgesic effects of the two agents were very weak when compared to radiotherapy (Hendriks et al., 2016). Recently, the multiple roles of the RANK-RANKL pathway in cancer have been emphasized. Higher RANK expression in primary breast cancer was associated with chemotherapy sensitivity and a higher risk of recurrence and death. Furthermore, the RANK-RANKL pathway stimulated breast cancer bone and lung metastasis (Tan et al., 2011; Pfitzner et al., 2014). Denosumab promoted apoptosis and decreased metastasis by inhibiting RANK-mediated mitogenic signaling of female hormones and abrogating RANK-induced increased mitochondrial respiration (Deligiorgi and Trafalis, 2020). It also disrupted the interaction between the tumor and the bone microenvironment (Scagliotti et al., 2012). Overactive cAMP/PKA signaling caused by Prkar1a gene deletion in aggressive osteosarcoma was responsible for the high expression of RANKL. Blocking RANKL inhibited osteosarcoma progression and lung metastasis in genetically engineered mouse models, and RANKL inhibitors even prevented osteosarcoma from occurring when utilized for pretreatment (Chen et al., 2015). Therefore, the anticancer potential of targeting RANKL-RANK makes denosumab a potential candidate for repurposing. However, more large-scale clinical trials are still needed to confirm its efficacy and safety.

### Other functions of the RANK/RANKL/OPG pathway

Now that the central role of the RANK/RANKL/OPG system in bone metabolism has been better understood, attention has turned to the multiple functions of this system and other therapeutic potentials of denosumab. Epidemiological studies found a positive correlation between the increase in OPG level and the increase in coronary calcification score, which might represent the regulatory mechanism of the body to fight against the disease. Denosumab inhibited aortic calcium deposition in RANKL knock-in mice, suggesting that OPG played a protective role (Samelson et al., 2014). In addition, studies in sarcopenia found that RANKL activated NF-kB signaling to induce insulin resistance in muscle cells in response to TNF-a, and recombinant human osteoprotegerin (OPG-Fc) reversed the resistance, increased glucose uptake in muscle cells, and downregulated the expression of inflammatory and anti-myogenic genes. Clinically, PMOPs who received chronic denosumab injections demonstrated improved muscle mass (Bonnet et al., 2019). Notably, malignancy-related hypercalcemia significantly reduced median survival. Denosumab treatment reduces serum calcium-correcting protein and significantly delays the time to first hypercalcemia effectively and durably in the majority (64%) of these PMOPs who were not sensitive to bisphosphonate therapy (Rosner and Dalkin, 2012; Hu et al., 2014). Furthermore, BRCA1 mutations in breast cells can autonomously activate RANKL expression and trigger breast cancer susceptibility. At the same time, denosumab can interfere with the crosstalk between RANKL-producing “sensor” cells and RANK+ “tumor-initiating responder cells.” Interestingly, metformin synergistically increased the sensitivity of this type of breast cancer to denosumab (Cuyàs et al., 2017). In addition, denosumab exhibited good tumor-suppressive effects on aneurysmal bone cysts with high RANKL expression in the tumor stroma (Palmerini et al., 2018). In the future, more combination chemotherapy strategies involving denosumab may emerge for RANKL-related tumors.

### The application of denosumab in the prevention of fractures

The long-term use of anti-osteoporotic drugs to prevent fractures has always been controversial. In addition to PMOP, denosumab was indicated for the subtype of osteoporosis, such as men with an increased risk of fractures. A meta-analysis showed that denosumab improved vertebral and non-pyramidal BMD significantly better than first-line fracture prevention bisphosphonates and was more durable during half-year and one-year treatment periods (Lyu et al., 2019). Currently, many PMOP patients are treated with combination or sequential therapies. The long-term combination of teriparatide and denosumab improved bone microarchitecture more than monotherapy, especially in cortical bone (Tsai et al., 2016). BMD was found to continuously increase when transitioning from teriparatide to denosumab, whereas transitioning from denosumab to teriparatide resulted in transient or temporary bone loss (Leder et al., 2015). In addition, follow-up of denosumab after a course of romosozumab increases bone mineral density steadily (Lewiecki et al., 2019). Non-pyramidal fractures were mainly caused by increased cortical fragility. The deposition process is required for the efficacy of bisphosphonates. The adsorption area of ​​bisphosphonates by cortical bone units was much smaller than that of cancellous bone trabeculae. In contrast, denosumab combined with RANKL was distributed in the bone uniformly and widely, allowing denosumab to reduce cortical porosity early and effectively and inhibit intracortical remodeling (Zebaze et al., 2014). Clinically, denosumab can effectively inhibit periprosthetic bone resorption and increase periprosthetic bone mineral density after a hip replacement (Aro et al., 2019). However, if we want to effectively control the prosthesis displacement and speed up functional recovery, more studies are needed to optimize the perioperative drug regimen. Of note, prior ZA use and current long-term use of denosumab were the risk factors for atypical femoral fractures (AFFs) (Takahashi et al., 2020). The possible reason was that the inhibition of osteoclasts over-inhibits bone remodeling, which made it challenging to repair microcracks in the femoral cortex caused by continuous loading. The pathology of AFFs is complex, and it is worth exploring the role of denosumab in it.

### Limitations

Herein, we improved the generalizability of bibliometric results by searching different databases and using multiple tools. However, there are some noted limitations. First, literature that fits the theme to a low degree was likely included when obtaining comprehensive data. Second, the early results have received more attention due to the longer publication time, resulting in insufficient attention to new hotspots and frontiers. Due to the limited space, there is no further comparison of their respective research advantages after highlighting the analysis of different results. Furthermore, we did not compare the literature types, including the meta-analysis, clinical trials, and clinical observation, because the bibliometrics did not focus on the different types of articles. Despite the noted limitations, this article presents denosumab’s research status and future trends comprehensively and objectively.

## Conclusion

The United States is a leader in the denosumab field. The achievements and influence of Amgen far exceed other institutions, but cooperation among high-yield countries and institutions is lacking. The clinical comparison and combination of denosumab with other drugs in treating OP was the primary topic for an extended period, followed by preventing defects and adverse events in the process of SREs. Multi-center large-sample long-term trials were the mainstream research methods. Among the recently emerged topics, the risk of denosumab withdrawal and related treatment strategies have brought the most attention and controversy. Meanwhile, the emergence of the new monoclonal antibody romosozumab and anabolic agent abaloparatide has also attracted the attention of many researchers. Exploring the multiple roles of the RANK-RANKL-OPG system in tumor progression, metastasis, and other diseases may be a potential direction for the basic research of denosumab in the future. In addition, optimizing the perioperative dosing schedule of denosumab for internal fixation and prosthesis implantation are also valuable clinical topics (Supplementary Table S2). Despite the extensive advantages and considerable cost-effectiveness of denosumab, there were still drawbacks, such as ONJ, severe hypocalcemia, high recurrence rate, and rare sarcoma transformation in treating GCTB, AFFs, etc., which needed to be solved.

## Data Availability

The original contributions presented in the study are included in the article/Supplementary Material; further inquiries can be directed to the corresponding author.
